# *Listeria monocytogenes* in the seafood industry: Exploring contamination sources, outbreaks, antibiotic susceptibility and genetic diversity

**DOI:** 10.1002/mbo3.70003

**Published:** 2024-10-17

**Authors:** Karlene Lambrechts, Diane Rip

**Affiliations:** ^1^ Department of Food Science Stellenbosch University Matieland South Africa

**Keywords:** fish, food safety, persistence, salmon, trout

## Abstract

Fish and seafood are rich sources of protein, vitamins, and minerals, significantly contributing to individual health. A global increase in consumption has been observed. *Listeria monocytogenes* is a known problem in food processing environments and is found in various seafood forms, including raw, smoked, salted, and ready‐to‐eat. Without heat treatment and given *L. monocytogenes*' ability to multiply under refrigerated conditions, consuming seafood poses a substantial health hazard, particularly to immunocompromised individuals. Numerous global outbreaks of listeriosis have been linked to various fish products, underscoring the importance of studying *L. monocytogenes*. Different strains exhibit varying disease‐causing abilities, making it crucial to understand and monitor the organism's virulence and resistance aspects for food safety. This paper aims to highlight the genetic diversity of *L. monocytogenes* found in fish products globally and to enhance understanding of contamination routes from raw fish to the final product.

## Graphical abstract

*Listeria monocytogenes* presents a significant concern in fish and its processing environments. This literature review aimed to gather global insights on fish and its processing settings, exploring genetic diversity and contamination pathways from raw fish through processing to distribution. Additionally, understanding the factors contributing to the persistence of *Listeria* in fish factories was a key objective.

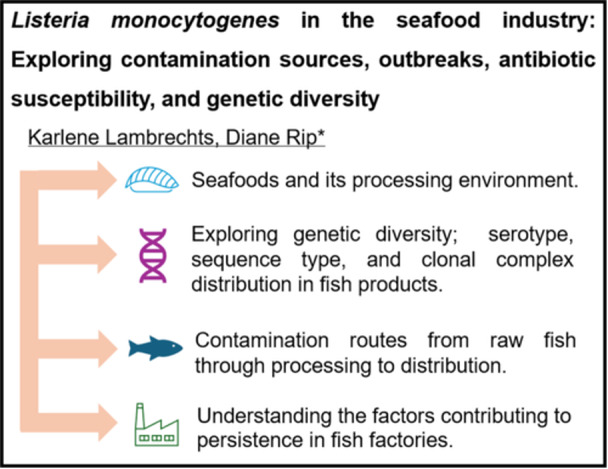

## 1 INTRODUCTION

*Listeria monocytogenes* holds significance in global public health and economic spheres (Matle et al., 2019). It can grow at temperatures ranging between 1°C and 50°C, can withstand freezing temperatures and is inactivated at 60°C for 30 min (Azizoglu et al., 2009; Batt, 2014). It can grow over a wide pH range (4–9.5), in low water activity (*a_w_* < 0.9) and in high sodium chloride concentrations (10%) (Abdollahzadeh, Ojagh, Hosseini, Ghaemi, et al., 2016; Schulz et al., 2023). *L. monocytogenes* can adapt to challenging environmental conditions associated with osmotic stress, cold stress, low pH, and desiccation (Kaszoni‐Rückerl et al., 2020). *L. monocytogenes* can grow in a range of environments containing a variety of these stresses, making this pathogen a particular concern in ready‐to‐eat (RTE) foods consumed without heating or cooking. Foods associated with *L. monocytogenes* include RTE foods, minimally processed foods, seafood, dairy products, red meat, poultry and fresh produce (Kathariou, 2002; Tchatchouang et al., 2020).

With growing human interest in healthy living, an increase in global seafood consumption (including raw seafood) can be observed (Figueieredo, 2022). Globally, fish consumption has already doubled since 1998 but is projected by Naylor et al. (2021) to increase further by 80% between 2015 and 2050. With no heat treatment and the ability of *L. monocytogenes* to survive and grow at refrigeration temperatures, fish, crustaceans, cold‐smoked salmon and other seafood can pose a significant risk to the health of consumers when eaten raw (Alvarez‐Molina et al., 2021; Chen et al., 2016; Hicks, 2016; Leong et al., 2015). *L. monocytogenes* thus poses a substantial challenge in producing safe food and is of significant concern (Braga et al., 2017).

Between 2010 and 2011, the European Union (EU) performed a baseline survey determining the prevalence of *L. monocytogenes* in food products at retail outlets. Of the 13 088 food samples tested, there was a prevalence of 10.4% in the smoked and gravad fish samples, followed by 2.07% in meat products and 0.47% in cheese (Matle et al., 2020). This highlights fish products as significant vehicles of *L. monocytogenes* contamination to consumers.

In the South African context, the average person (in 2017) ate 5.97 kg of fish per year of which 50% was caught locally; 70% of locally caught fish were sardines and hake. Higher‐income South Africans consumed more speciality fish lines such as salmon, sole, kingklip portions, scallops or ready‐crumbed prawns (Figueieredo, 2022). The population also has a very high proportion of vulnerable individuals with immunocompromising infections such as human immunodeficiency virus (HIV), acquired immunodeficiency syndrome (AIDS), diabetes and tuberculosis (TB), putting them at higher risk for listeriosis (Matle et al., 2019). Listeriosis is a notifiable human disease in numerous countries, including South Africa.

Globally, *L. monocytogenes* is reported from food processing environments and in fish: raw, smoked and salted. This review explores the global distribution and genetic characteristics of *L. monocytogenes* in seafood. By understanding contamination sources, potential risks can be identified and mitigated. Given *L. monocytogenes*' link with RTE foods, an increase in genomic data outputs is very important and helpful for epidemiological purposes.

## 2 GENETIC DIVERSITY

### 2.1 Lineage groups and serotypes

Serotyping is a first‐level subtyping method used for epidemiological purposes and was the first *L. monocytogenes* subtyping scheme. It is a typing method based on the presence of somatic (O) and flagellar (H) antigens (Allerberger, 2003; Capita et al., 2019; Hyden et al., 2016). Fifteen possible O antigens and four possible H antigens occur, and the serogroups are assigned based on the unique combination. *L. monocytogenes* was until recently classified into 13 serotypes (Allerberger, 2003; Jami et al., 2014; Jamshidi & Zeinali, 2019). Recent studies employed methods to rapidly characterize a novel serotype 4h (hybrid sublineage II) displaying hypervirulent properties (Feng et al., 2020). Different serotypes are associated with different environments or ability to cause disease (Feng et al., 2020; Jamshidi & Zeinali, 2019).

*L. monocytogenes* is further categorized into four genetic lineages (I–IV), with each lineage comprised of distinct serotypes (Abdollahzadeh, Ojagh, Hosseini, Irajian, et al., 2016; Matle et al., 2020). The majority of *L. monocytogenes* isolates fall into lineages I and II (Orsi et al., 2011). Lineage I show a preference for human and animal tissue and cell types, while lineage II is more adaptive to environmental conditions (Kaszoni‐Rückerl et al., 2020). Human listeriosis cases are mostly linked to serotypes 4b, 1/2b and 1/2a (Alvarez‐Molina et al., 2021; Batt, 2014; Hyden et al., 2016; Jami et al., 2014). Additionally, serotype 1/2a is mostly isolated from food (Basha et al., 2019; Matle et al., 2020; Orsi et al., 2011). Serotype 4b is more frequently isolated from clinical cases than serotypes 1/2a and 1/2c relative to its frequency in food and the food processing environment (Maury et al., 2016). Food items are typically linked with *L. monocytogenes* characterized by reduced pathogenicity and virulence (Maury et al., 2016). A reason that some serotypes are more associated with clinical cases is their virulence and ability to adapt to the human host (Matle et al., 2020). The antigenic group 1/2 (1/2a, 1/2b and 1/2c) has been overrepresented in food isolates and is also increasing in human clinical cases (Wagner et al., 2022). Table 1 illustrates the distribution of *L. monocytogenes* lineages and serotypes in seafood and its food processing environment, reported globally.

**Table 1:** Lineages and serotypes of *Listeria monocytogenes* reported from seafood and its processing environment globally.

Lineage type	Serotype	Origin	Country	Year	Method	Reference
I	1/2b, 4b	Fish (not specified), smoking room drain, smoked trout roulade, fresh hake	South Africa	2018–2021	WGS	Lambrechts et al. (2024)
	N/A	Raw seafood	South Africa	2020	PCR‐RFLP	Keet and Rip (2021)
	1/2b	RTE tilapia sashimi final product and processing plant (workers' gloves and facility surfaces)	China	2016	Commercial serotyping kit	(Chen et al. (2016))
	1/2b	Raw rainbow trout	Finland	2000	Antisera against O and H antigens	Miettinen and Wirtanen (2005)
	1/2b	RTE seafood	Korea	2017	*Listeria* antisera set	Lee et al. (2017)
	1/2b	RTE seafood food processing environment	Korea	2017	*Listeria* antisera set	Lee et al. (2017)
	3b	RTE tilapia sashimi final product and processing plant (workers' gloves and facility surfaces)	China	2016	Commercial serotyping kit	Chen et al. (2016)
	4b	Frozen food	Montevideo‐ Uruguay	2011–2013	Multiplex PCR	Braga et al. (2017)
	4b	RTE tilapia sashimi final product and processing plant (workers' gloves and facility surfaces)	China	2016	Commercial serotyping kit	Chen et al. (2016)
	4b	RTE smoked salmon from retailer	Spain	2011	*Listeria* antisera set	González et al. (2013)
	4b	RTE cold‐salted and cold‐smoked rainbow trout and processing line and surroundings (skinning)	Finland	1996–1997	Agglutination method	Johansson et al. (1999)
	4b	RTE seafood	Korea	2017	*Listeria* antisera set	Lee et al. (2017)
	4b	Raw seafood	Iran	2012–2014	Commercially prepared *Listeria* antisera set	Jamali et al. (2015)
	4e	RTE tilapia sashimi final product and processing plant (workers' gloves and facility surfaces)	China	2016	Commercial serotyping kit	Chen et al. (2016)
	4b, 4e	Smoked and unsmoked fish and/or processed shellfish processing facilities (swab: pooled water)	Republic of Ireland	2013–2014	Multiplex PCR and antisera testing	Leong et al. (2015)
	4b, 4d, 4e	Fresh fish (freshwater) and smoked fish (marine)	Poland	2014–2016	Multiplex PCR	Wieczorek and Osek (2017)
	1/2b, 3b, 7	Raw mackerel	India	2015‐2018	Multiplex PCR	Basha et al. (2019)
	1/2b, 3b, 7	Caspian tyulka (raw fish) at the retail market and tilapia fillet (raw fish) at a processing facility	Iran	2017	Multiplex PCR	Abdollahzadeh, Ojagh, Hosseini, Irajian, et al. (2016)
	1/2b, 3b, 7	Fresh fish (marine and freshwater), smoked fish (marine and freshwater)	Poland	2014–2016	Multiplex PCR	Wieczorek and Osek (2017)
II	N/A	Raw seafood	South Africa	2020	PCR‐RFLP	Keet and Rip (2021)
	1/2a	Salmon ribbon, cold smoked trout ribbon, smoked trout terrine, oysters, fresh hake, fish room floor and salmon room drain	South Africa	2018‐2021	WGS	Lambrechts et al. (2024)
	1/2a	Frozen food	Montevideo‐ Uruguay	2011–2013	Multiplex PCR	Braga et al. (2017)
	1/2a	Smoked fish, unsmoked fish and or processed shellfish processing facilities (swab: drains, box, floors, shelves, tables), raw salmon, smoked salmon	Republic of Ireland	2013–2014	Multiplex PCR and antisera testing	Leong et al. (2015)
	1/2a	RTE smoked salmon from retailer	Spain	2011	*Listeria* antisera set	González et al. (2013)
	1/2a	RTE cold‐smoked, cold‐salted rainbow trout, hot‐smoked fish	Finland	1996–1997	Agglutination method	Johansson et al. (1999)
	1/2a	RTE seafood	Korea	2017	*Listeria* antisera set	Lee et al. (2017)
	1/2a	Raw fish	Iran	2012–2014	Commercially prepared *Listeria* antisera set	Jamali et al. (2015)
	1/2a	Open‐air fish market (hands, knives, work surfaces, containers)	Iran	2012–2014	*Listeria* antisera set	Jamali et al. (2015)
	1/2a	Processing line and surroundings (skinning, salting, packing, drain, slicing)	Finland	1996	Agglutination method	Johansson et al. (1999)
	1/2c	Meat processing facility	Spain	2017	WGS‐based MLST	Alvarez‐Molina et al. (2021)
	1/2c	Frozen food	Montevideo‐ Uruguay	2011–2013	Multiplex PCR	Braga et al. (2017)
	1/2c	Raw fish	Iran	2012–2014	*Listeria* antisera set	Jamali et al. (2015)
	1/2a, 3a	Fresh fish (marine and freshwater), smoked fish (marine)	Poland	2014–2016	Multiplex PCR	Wieczorek and Osek (2017)
	1/2a, 3a	Raw mullet and retail ice	India	2015–2018	Multiplex PCR	Basha et al. (2019)
	1/2a, 3a	Caspian tyulka (raw fish) at the retail market and tilapia fillet (raw fish) processing facility and rainbow trout (raw) at the retail market	Iran	2017	Multiplex PCR	Abdollahzadeh, Ojagh, Hosseini, Irajian, et al. (2016)
	1/2c, 3c	Fresh fish (marine)	Poland	2014–2016	Multiplex PCR	Wieczorek and Osek (2017)

Abbreviations: MLST, multilocus sequencing typing; PCR‐RFLP, polymerase chain reaction‐restriction fragment gel electrophoresis; WGS, whole‐genome sequencing.

In a South African study, lineage II was the most prevalent (*p* < 0.01) among isolates from seafood and its processing environment (*n* = 38) (Lambrechts et al., 2024). A similar trend of lineage II in seafood and its processing environment has been reported by researchers globally (Iran, India, Poland, France, and Norway) (Abdollahzadeh, Ojagh, Hosseini, Ghaemi, et al., 2016; Fagerlund, Idland et al., 2022; Maury et al., 2019; Wieczorek & Osek, 2017; Wieczorek et al., 2020). The opposite was also reported with a majority of lineage I isolates found in seafood and its processing environment from Finland, Korea and South Africa (Table 1) (Keet & Rip, 2021; Lee et al., 2017; Miettinen & Wirtanen, 2005). The study conducted in the Western Cape, South Africa, on locally sourced fish (raw hake) categorized isolates only into lineage groups (Keet & Rip, 2021). It found that lineage I comprised a significantly higher proportion of the raw hake samples (95%) compared to lineage II. However, these results were derived from multiple sampling occasions at a single factory.

From the literature, the predominant serotype isolated in seafood and its processing environment is serotype 1/2a followed by serotypes 4b, 1/2b and 1/2c (Table 1). Seafood contaminated with *L. monocytogenes* lineage I, serotype 4b, originated from various types of fish, with many associated with RTE fish products in countries such as China, Korea, Spain, Finland, Ireland, Iran and South Africa.

### 2.2 Sequence types and clonal complexes

Ninety‐five per cent of invasive listeriosis infections are caused by three serotypes (4b, 1/2b, and 1/2a), stressing the importance of greater strain determination. The four genetic lineages and subsequent serotypes of *L. monocytogenes* can be further subdivided into sequence types (STs) and clonal complexes (CCs). STs are categorized based on seven housekeeping genes and depending on the variation of the nucleotide sequences of these genes, an ST is assigned (Enright et al., 2000, 2002; Seidl et al., 2015). MLST describes population structures and the phylogeny of many bacterial pathogens (Painset et al., 2019). STs can be grouped into CCs, with every ST in that group sharing at least five of the seven identical alleles with one other ST in that group (Enright et al., 2000, 2002; Seidl et al., 2015).

Some clones and STs are more associated with the food processing environment and others more with clinical cases. These different host tropisms are not fully understood yet and can be linked to virulence potential or the ability to adapt to harsh environmental conditions, allowing for multiplication, persistence and survival (Fagerlund, Wagner, et al., 2022). Although research shows that some STs are more pathogenic than others, the food safety and regulatory authorities consider all isolates to display the same risk from a food safety point of view (Wagner et al., 2022).

Table 2 shows the CCs of *L. monocytogenes* in seafood and its food processing environment that other researchers reported globally. In a study done in Poland by Wieczorek et al. (2020), CC121 (ST121) was isolated from most fish (cold‐smoked salmon and raw salmon) and its processing environment, followed by CC8 (ST8). The authors also found that 96% of these isolates were from lineage II (Table 2).

**Table 2:** Clonal complexes of *Listeria monocytogenes* reported from seafood and its processing environment globally.

Clonal complex	Origin	Number of isolates	Country	Year	Method	Reference
CC1, CC3, CC5, CC54, CC87, CC121, CC155, CC204	Seafood and food processing environment	*N* = 16	South Africa	2018–2021	WGS	Lambrechts et al. (2024)
CC1, CC2, CC3, CC7, CC8, CC11, CC14, CC18, CC19, CC20, CC31, CC37, CC88, CC91, CC121, CC177, CC315, CC403, CC415 (76% Lineage 2)	Salmon food processing environment (environmental, gutted salmon, packaged salmon, raw materials entering)	*N* = 306	Norway	1990–2020	WGS	Fagerlund, Wagner, et al. (2022; Wagner et al. (2022)
CC1, CC2, CC3, CC4, CC5, CC6, CC7, CC9, CC11, CC14, CC87, CC88, CC101, CC121, CC155, CC199, CC288, CC321, CC573	Seafood and food processing environment	*N* = 209	United States of America	1999–2019	WGS	Daeschel et al. (2022)
CC1, CC2, CC3, CC5, CC6, CC7, CC9, CC14, CC18, CC20, CC31, CC37, CCCC59, CC121, CC124, CC155, CC193, CC199, CC204, CC218, CC288, CC321, CC412, CC451, CC475	Seafood and food processing environment	*N* = 406	France	2005–2016	WGS	Maury et al. (2019)
CC1, CC2, CC3, CC5, CC6, CC7, CC8, CC9, CC14, CC20, CC31, CC88, CC155, CC193, CC199, CC321, CC415	Fish and seafood	*N* = 148	Canada	N/A	WGS	Cooper et al. (2021)
CC1, CC2, CC3, CC5, CC6, CC7, CC8, CC9, CC14, CC21, CC31, CC37, CC59, CC87, CC101, CC121, CC155, CC204	Fish and fishery products	*N* = 223	EU	2010–2011	WGS	Painset et al. (2019)
CC11, CC121, CC199, CC321	Cold‐smoked fish and the food processing environment	*N* = 42	N/A	1998–2015	WGS	Harrand et al. (2020)
CC1, CC7, CC8, CC9, CC31, CC121, CC155, CC475 (96% lineage 2)	Raw and cold‐smoked salmon and the food processing environment	*N* = 28	Poland	2019	WGS	Wieczorek et al. (2020)
ST1, ST2, ST3, ST5, ST6 ST8, ST9, ST59, ST87, ST121, ST155, ST299, ST705^a^	Fish foods	*N* = 133	China	2010–2019	WGS	Cheng et al. (2022)

^a^ Sequence types (STs) and not clonal complexes (CCs) were reported in this study.

Painset et al. (2019) found that the most prevalent CC in fish products and their food processing environment in various countries in the EU was CC121, followed by CC8, CC9 and CC155. Maury et al. (2019) found similar results in France, with CC121 being the most prevalent in seafood, followed by CC9. Several researchers also found CC321 to be prevalent in seafood in the United States and Canada, among other countries (Cooper et al., 2021; Daeschel et al., 2022; Harrand et al., 2020). Fagerlund, Idland, et al. (2022) examined Norwegian clinical isolates, and ST121 (CC121) was the second most prevalent (13%) ST found within the clinical isolates examined (Table 2). The authors also found that 80% of the clinical isolates examined belonged to lineage II. ST121 is considered hypovirulent; however, the relatively high prevalence of clinical cases attributed to these strains in Norway shows their ability to cause disease (Fagerlund, Idland, et al., 2022). A study characterized *L. monocytogenes* from fish and fish‐processing environments in Poland. Authors reported CC6 and CC121 to be the most prevalent (Zakrzewski et al., 2023). Thus far, one study in South Africa has categorized *L. monocytogenes* isolates from locally caught seafood and its food processing environment into serotype, ST or CC using WGS (Lambrechts et al., 2024). The authors reported ST3, ST5, ST54, ST87, ST121, ST155, ST204 and ST515 over their subset of isolates.

## 3 LISTERIOSIS AND OUTBREAKS

Human listeriosis is caused by *L. monocytogenes* and can present in an invasive or noninvasive form with a 90% hospitalization rate and a mortality rate of 20%–30% (in the immunocompromised) (Camejo et al., 2011; Hernandez‐Milian & Payeras‐Cifre, 2014). The European Food Safety Authority (European Food Safety Authority, 2021) reported a fatality rate of 13% in 2020. *L. monocytogenes* can cause infections in immunocompromised and immunocompetent individuals but especially causes problems in pregnant women, newborns, the elderly and immunosuppressed individuals (Camejo et al., 2011; Liu, 2006; Orsi & Wiedmann, 2016). Increased susceptibility to listeriosis in immunosuppressed individuals includes those undergoing cancer or immunosuppressive therapies or individuals who had organ transplants (Lungu et al., 2011).

Consumption of contaminated food is the main route of infection with transmission from infected animals to humans being very rare (Allerberger & Wagner, 2010; Chen et al., 2016). After consumption, the organism can pass through the epithelium lining of the gastrointestinal tract (Leong et al., 2015). What further complicates the situation is the uncertainty around the infectious dose of *L. monocytogenes*. It can be as low as <100 CFU/g (Jeyasanta & Patterson, 2016). Important factors playing a role in the infectious dose include the amount and type of food consumed, the number of pathogens ingested and their virulence, and the host's immune response (Matle et al., 2019; Wagner et al., 2022). Furthermore, the incubation period of listeriosis is between 1 and 70 days, making the source of infection difficult to identify during outbreaks or in sporadic cases (Goulet et al., 2013; Johansson et al., 1999).

Noninvasive listeriosis develops mostly in immunocompetent individuals with febrile gastroenteritis and flu‐like symptoms (Camejo et al., 2011; Kayode & Okoh, 2020). Symptoms include fever, headache, diarrhea and muscle pain and occur after the ingestion of contaminated food (Food Safety Authority of Ireland, 2011; Kayode & Okoh, 2020). The symptoms of noninvasive listeriosis are self‐limiting, with individuals feeling well in a short timeframe without needing to seek medical attention. Because of this, it is often undiagnosed and thus underreported (Matle et al., 2020).

Invasive listeriosis is associated with infection in blood, liver or cerebral fluid (considered the sterile parts of the body) (Zhu et al., 2017). This form mostly targets immunocompromised individuals (Camejo et al., 2011; Food Safety Authority of Ireland, 2011). Invasive listeriosis can manifest as septicemia, meningitis, encephalitis, meningoencephalitis or intrauterine infections. *L. monocytogenes* can cross the fetoplacental barrier, allowing infection of the fetus from the infected mother with the possibility of spontaneous abortion or stillbirth (Camejo et al., 2011; Johnson et al., 2021).

Listeriosis associated with seafood is less frequently reported than listeriosis associated with other food products. However, various factors can potentially play a role in this: (1) *L. monocytogenes* is generally present in seafood in very low numbers, (2) small quantities of the product are ingested at once, (3) consumers are aware that seafood must be refrigerated, (4) lack of sufficient epidemiological tracking as a result of the long incubation period after eating contaminated food, and (5) decreased consumption of these high‐risk foods by immunocompromised individuals (Jami et al., 2014).

Foodborne illnesses and outbreaks related to seafood have been reported worldwide and are caused by various pathogens including bacteria, viruses and parasites (Chen et al., 2016). Table 3 summarizes some of the outbreaks related to *L. monocytogenes* in seafood. The three most recent outbreaks related to seafood were multicountry outbreaks from RTE fish products such as smoked trout/salmon, associated with ST155, ST394, and ST1247 respectively. Furthermore, many of the other outbreaks were also related to smoked fish, a product commonly consumed as RTE food.

**Table 3:** *Listeria monocytogenes* outbreaks related to seafood (adapted from Jami et al., 2014).

Product	Country	Year	Number of cases, deaths^a^	Serotype, ST, CC	Reference
RTE fish products	Multicountry	2019–2023	64 Cases, 10 deaths	ST155	EFSA (2023)
Smoked rainbow trout	Multicountry	2020–2021	55 Cases	ST394	Halbedel et al. (2018)
Cold‐smoked salmon and trout products	Multicountry	2014–2019	22 Cases, 5 deaths	ST1247 (CC8)	EFSA (2019)
Cold‐smoked fish	Denmark	2014–2015	10 Cases, 3 deaths	ST6	Gillesberg Lassen et al. (2016)
Herring cutlet marinated in oil	Germany	2010	8 Cases, 1 death	‐	Jami et al. (2014)
Vacuum‐packed fish	Finland	1999–2000	10 Cases, 4 deaths	1/2	Hatakka et al. (2000)
Smoked rainbow trout	Finland	1999	5 Cases	1/2a	Miettinen et al. (1999)
Tuna corn salad	Italy	1997	1566 cases	4b	Aureli et al. (2000)
Imitation crab meat	Canada	1996	2 Cases	1/2b	Farber et al. (2000)
Cold‐smoked rainbow trout	Sweden	1994–1995	9 Cases, 2 deaths	4b	Ericsson et al. (1997)
Smoked mussels	New Zealand	1992	3 Cases, 1 death	1/2a	Brett (1998))
Smoked mussels	Australia	1991	4 Cases	1/2a	Jami et al. (2014)
Shrimp	United States of America	1989	2 Cases	4b	Riedo et al. (1994)
Smoked cod roe	Denmark	1989	1 Case	‐	Jami et al. (2014)
Fish	Italy	1989	1 Case	‐	Facinelli et al. (1989)
Fish and molluscan shellfish	New Zealand	1980	22 Cases, 7 deaths	1b and 1/2a	Jami et al. (2014)

Abbreviations: CC, clonal complex; RTE, ready‐to‐eat; ST, sequence type.

^a^ Where reported.

## 4 CONTAMINATION AND SOURCES OF CONTAMINATION IN SEAFOOD

*L. monocytogenes* has been found in environmental sources such as soil, plant material, silage, fresh and salt water, feces of healthy animals and raw foods associated with these environments (Chen et al., 2016; Jami et al., 2014; Leong et al., 2014). Therefore, various routes for contamination of food and transmission to humans exist (Zhang et al., 2021). In addition to raw materials being contaminated in the food processing environment, personnel, floors, drains, racks and rollers are all sources of contamination (Fonnesbech Vogel et al., 2001; Zhang et al., 2021). RTE foods pose a risk for listeriosis in the absence of a microbial inactivation step, for example, heating (Leong et al., 2015).

Proper handling and heat treatment are often enough to inactivate these pathogens; however, the consumption of raw seafood has increased significantly over the past two decades (Chen et al., 2016). With no heat treatment and *L. monocytogenes* able to survive and proliferate at refrigeration temperatures and under low‐pH conditions, fish, crustaceans, molluscs, cold‐smoked salmon and other seafood can pose a significant risk to the health of consumers when eaten raw (Alvarez‐Molina et al., 2021; Chen et al., 2016; Leong et al., 2015). Furthermore, the ubiquitous nature of *L. monocytogenes*, its ability to form protective biofilms and its ability to grow under stress conditions contribute to its persistence in the food processing environment (Alvarez‐Molina et al., 2021; Zakrzewski et al., 2024).

Seafood can support the growth of *L. monocytogenes* (Leong et al., 2015), and it is therefore crucial to reduce the amount of *L. monocytogenes* in seafood to a minimum (Fonnesbech Vogel et al., 2001). The cold‐smoking process does not kill *L. monocytogenes* (Pearson et al., 2023). A challenge study done by Leong et al. (2015) found that cold‐smoked salmon (from two different retailers) could support the growth of *L. monocytogenes* under the test conditions. Cold‐smoked fish is described as lightly preserved whereby the fish undergoes processing by smoke at a temperature not high enough to result in complete coagulation of the proteins, and therefore only partial coagulation of the proteins takes place (Jahncke, 2008). Besides a higher smoking temperature and increased salt concentration (both contributing to undesirable organoleptic properties), no critical control point exists for the production of cold‐smoked fish products (Jahncke, 2008; Pearson et al., 2023). The minimal or lack of heat application during the production of cold‐smoked salmon presents various challenges to this industry (Harrand et al., 2020). While the extent of reduction varies among producers, determining the exact reduction through this process is challenging and a level of risk to the consumer remains (Leong et al., 2015). Furthermore, cold‐smoked products are extensively handled after processing, providing several opportunities for cross‐contamination of *L. monocytogenes* and other pathogens. These fish products are typically eaten as RTE products without prior heating (Jahncke, 2008).

*L. monocytogenes* can be isolated from various seafoods, and contamination can result from a variety of routes such as the aquaculture environment, the harvesting process, transportation tanks, handling and the processing environment (Chen et al., 2016). Understanding the contamination routes of *L. monocytogenes* in the fish processing industry is important to prevent contamination of the final product.

### 4.1 Contamination of raw incoming fish

Various studies have determined the route of contamination of *L. monocytogenes* in a seafood processing facility and have shown that contamination of the final product does not always originate from the raw incoming fish. The prevalence of *L. monocytogenes* in raw fish varies from 0% to 10% but is generally quite low, with a higher probability of contamination in fish sourced from water receiving a heavy runoff from land (Chen et al., 2016; Fonnesbech Vogel et al., 2001; Miettinen & Wirtanen, 2005).

A systematic review and meta‐analysis detailing the prevalence of *Listeria* and *L. monocytogenes* prevalence in fish, fish products and the fish processing environment, reported a prevalence of 5.8% *L. monocytogenes* from raw fish versus a 14.5% prevalence in ready‐to‐eat products (Zakrzewski et al., 2024). The study also found a significantly higher prevalence of *L. monocytogenes* in raw fish in high‐income countries (15%) compared to 3.0% in lower to middle‐income countries. This study further categorized *L. monocytogenes* from raw fish by family, with pooled results showing the highest contamination in *Pleuronectidae* and *Salmonidae*, at 21.4% and 28.5%, respectively (Zakrzewski et al., 2024). *Pleuronectidae* includes commercially important flatfish like halibut, and sole, typically found in cold and temperate waters, while *Salmonidae*, found in cold‐water environments mainly in the Northern Hemisphere, includes species such as salmon and trout. Additionally, species characterization in this study showed a 50% prevalence of *L. monocytogenes* in *Mugil cephalus*, the flathead gray mullet commonly found in coastal and surface waters of lakes, where *Listeria* is also commonly found (Zakrzewski et al., 2024). Consequently, contamination of processing facilities with this species may be linked to imported raw fish; the longer time between catching and sale enhances the detection rate (Zakrzewski et al., 2024). Further, species *Oncorhynchus mykiss* and *Salmo salar* were associated with prevalence rates of 36.9% and 30.3%, respectively (Zakrzewski et al., 2024), known as rainbow trout/steelhead and Atlantic salmon, respectively.

A study by Miettinen and Wirtanen (2005) on farmed rainbow trout found that most of the *L. monocytogenes* contamination was in the gills of the fish and only occasionally in the skin and viscera. Gills serve as an ideal environment for bacterial growth and are the first part of the fish to be contaminated whenever volumes of contaminated water filter through (Miettinen & Wirtanen, 2005). Knowledge of the location of *L. monocytogenes* on the fish is important for the development and choice of transportation, handling and processing methods to avoid *L. monocytogenes* contamination to the rest of the fish, such as the pure fish flesh (Miettinen & Wirtanen, 2005). Additionally, minimizing the contamination of raw fish is vital to prevent the contamination of processing equipment and surfaces (Miettinen & Wirtanen, 2005). Furthermore, Miettinen and Wirtanen (2006) established that the seasons and weather had a strong influence on the presence of *Listeria* spp. in fish farms. Fagerlund, Idland, et al. (2022) also verified that the presence of *L. monocytogenes* was positively correlated with rainfall.

Chen et al. (2016) found no contamination of transportation tanks, the aquaculture environment or raw fish samples before processing in an RTE tilapia sashimi processing plant. However, final products, workers' hands and facility surfaces were contaminated with *L. monocytogenes*. This suggested that contamination was most likely due to inadequate cleaning and sanitation processes rather than the aquaculture or transportation tanks.

### 4.2 Contamination of products from the food processing environment

Although raw incoming fish is an important and valid source of contamination, contamination from the food processing environment and equipment during processing of the fish and after processing of the final product is the main source of contamination (Chen et al., 2010, 2016; Miettinen & Wirtanen, 2006; Pagadala et al., 2012; Rørvik et al., 2000). Contamination from the food processing environment includes food contact surfaces such as trimming boards, slicing machines, conveyor belts, weighing tables, skinning units, salting units and smokehouses (Chen et al., 2010; Johansson et al., 1999; Rørvik et al., 2000). Fagerlund, Wagner, et al. (2022) found that drains, floors, conveyor belts and gutting machines were most commonly associated with *L. monocytogenes* in salmon processing facilities.

Lin et al. (2006) studied the survival and adherence of *L. monocytogenes* on surfaces of commercial slicers and found that *L. monocytogenes* could be transferred from the slicer onto deli meats. Cross‐contamination and recontamination of RTE food within the food processing environment are a common source of *L. monocytogenes* contamination (Pagadala et al., 2012). Chen et al. (2010) found a prevalence of *L. monocytogenes* in chiller water and on trimming boards, suggesting that these could be a source of cross‐contamination to fish fillets.

Preventing the introduction of *L. monocytogenes* into the food processing environment is a very important step in controlling *L. monocytogenes* in seafood production because contamination of the facility can lead to contamination of the food itself (Leong et al., 2015; Rørvik et al., 2000). Rørvik et al. (2000) found *L. monocytogenes* on pallets and floors of the loading vehicles at the transport terminal, concluding that this might be an important factor in contaminating the processing plants with *L. monocytogenes*.

The specific source of environmental contamination (food contact surfaces and nonfood contact surfaces) is difficult to identify within a processing plant, and contamination can come from many sources including raw material, personnel and packaging material (Pagadala et al., 2012).

### 4.3 Contamination during and after distribution from the food processing environment

A study done by Miya et al. (2010) showed that the cell count of *L. monocytogenes* in final seafood products increased considerably with temperature abuse over 2 days at 10°C. Inappropriate storage or temperature abuse can happen during distribution or before consumption.

In an EU‐wide baseline study between 2010 and 2012, 3053 smoked or gravad fish samples were tested for *L. monocytogenes* prevalence and cell counts at the retail level. At the time of sampling the fish products, 10.4% of products were positive for *L. monocytogenes* and at the end of shelf life, 10.3% of samples were positive for *L. monocytogenes*. Furthermore, 1.7% of these samples had a cell count higher than 100 CFU/g at the end of the shelf life (European Food Safety Authority, 2013).

A study done by González et al. (2013) in Spain found that all the RTE smoked salmon products bought in the retail market were at temperatures higher than the recommended 4°C. Furthermore, the temperature of the smoked salmon was on average 8.0°C (5–14°C) (González et al., 2013). The authors also observed that when transported using regular/plastic shopping bags, the product's temperature could increase by an average of 6.2°C on the way home (González et al., 2013). Garrido et al. (2010) found that most consumers were unaware of the temperature of their refrigerators and that many consumers stored their RTE products at temperatures above 6°C. Based on this information, the consumer has a responsibility for the proper handling and storage of RTE food. Education and communication with the consumer about appropriate practices for buying, transporting and storing these high‐risk foods are important (González et al., 2013).

It is difficult to produce fish products, in particular cold‐smoked salmon, completely *L. monocytogenes* free. Therefore, it is important to introduce food safety programs at the preprocessing and processing steps. Additionally, new disinfectants must be incorporated to control persistent *L. monocytogenes* strains that could have adapted to the routine disinfectants used (Mahgoub et al., 2022). According to the guidelines of the European Salmon Smokers Association, the standard for *L. monocytogenes* is that it must be absent in 25 g of food before it has left the immediate control of the food processing environment. If *L. monocytogenes* is present in the final product, the food business operator may set intermediate limits if they can prove that the product will not exceed the limit of 100 CFU/g over the shelf life of the product (European Salmon Smokers Association, 2018).

Many studies reported higher frequencies of *Listeria* spp. over *L. monocytogenes* (Pagadala et al., 2012). The detection of any *Listeria* spp., however, is an indication of poor sanitation and cleaning practices and indicates the high probability that *L. monocytogenes* will be present sooner or later (Cocolin et al., 2002; Cossu et al., 2016).

### 4.4 Persistence

Once *L. monocytogenes* enters a food processing facility, it is very difficult to eradicate if no proper monitoring programs are in place. The survival of *L. monocytogenes* is influenced by several complex factors, and it can grow under stress conditions, as noted earlier. Persistent strains can be defined as a specific strain of *L. monocytogenes* isolated repeatedly from the same environment over an extended time (6 months and longer) (Leong et al., 2015). The presence of persistent strains increases the possibility of cross‐contamination of final food products as these strains are not eliminated from the food processing environment (Palaiodimou et al., 2021). Several cases of persistent strains have been documented in seafood processing environments (Chen et al., 2016; Fonnesbech Vogel et al., 2001; Palma et al., 2020).

Several factors can result in the persistence of *L. monocytogenes* in food processing environments. Harborage sites such as cracks, the inside of equipment and areas protected from cleaning and sanitation are areas where the sanitizer cannot reach properly and as a result favor the proliferation and growth of *L. monocytogenes* (Møretrø et al., 2017; Palaiodimou et al., 2021). This persistence is further complicated by various studies showing that *L. monocytogenes* displays tolerance to sanitizers commonly used in the food processing environment (Matle et al., 2019). The formation of biofilms in food processing environments contributes to *L. monocytogenes*' sanitizer tolerance (Møretrø & Langsrud, 2004; Rip & Gouws, 2020).

Persistent strains of *L. monocytogenes* can also be present due to the introduction or adaptation of specific strains to the selection pressures within the food processing environment. Suboptimal conditions (temperatures and disinfection concentrations) within the food processing environment can promote the survival and multiplication of these strains (Larsen et al., 2014; Palma et al., 2020). These adaptations (i.e. sanitizer tolerance genes or stress survival genes) may protect the isolates from the sanitizers or stresses encountered in the food processing environment (Palma et al., 2020). Persistence can indicate inadequate cleaning and sanitation processes or inadequate implementation thereof. Staff should be properly trained to prevent cross‐contamination of *L. monocytogenes* from the environment to products, ensuring a safe product (Chen et al., 2016; Palaiodimou et al., 2021; Palma et al., 2020). ST121 has been shown in many studies to be able to persist in the food processing environment for months or even years (Fagerlund, Wagner, et al., 2022; Harter et al., 2017; Holch et al., 2013; Maury et al., 2016; Myintzaw et al., 2023; Painset et al., 2019; Pasquali et al., 2018). CC121 has been the most common CC identified in many seafood factories (Maury et al., 2019; Painset et al., 2019; Wagner et al., 2022; Wieczorek et al., 2020), and persisting in factories in many European countries (Holch et al., 2013; Maury et al., 2016; Myintzaw et al., 2023; Painset et al., 2019). CC1 has been reported as one of the most common CCs in a salmon processing facility in Norway and has also been found to be persistent in these factories (Fagerlund, Wagner, et al., 2022; Wagner et al., 2022). Furthermore, ST3 (CC3) has also been found to persist in two king oyster mushroom production plants (Xu et al., 2023).

## 5 ANTIBIOTIC TREATMENT AND RESISTANCE

*L. monocytogenes* can invade almost any cell type, which can make the treatment of human listeriosis quite difficult. The incubation period of *L. monocytogenes* can range from 1 to 70 days (30 days average) and thus, the duration of treatment may vary depending on the level of infection (Goulet et al., 2013; Jeyasanta & Patterson, 2016; Olaimat et al., 2018). The outcome of listeriosis infection is dependent on early administration of antibiotics as *L. monocytogenes* is susceptible to most of the clinically relevant antibiotics used, except for fosfomycin, cephalosporin and fluoroquinolones, for which it has intrinsic resistance (Matle et al., 2019).

Ampicillin or penicillin G with an aminoglycoside (e.g. gentamicin) is the first line of therapy for human listeriosis (Grosboillot et al., 2022; Park et al., 2022). The second choice of therapy is trimethoprim in combination with a sulfonamide (such as sulfamethoxazole‐co‐trimoxazole). The combination is usually used when an individual is allergic or sensitive to β‐lactams or in cases of resistance to β‐lactams. Additionally, tetracycline, erythromycin and vancomycin can also be used to treat human listeriosis (Abdollahzadeh, Ojagh, Hosseini, Ghaemi, et al., 2016; Matle et al., 2019). Erythromycin is an antimicrobial agent of choice to treat pregnant women with listeriosis (Wieczorek & Osek, 2017).

Antimicrobial resistance of *L. monocytogenes* is increasing and is linked to the overuse of antibiotics in the clinical setting as well as the heavy use thereof in farm animals to promote growth (Matle et al., 2020). Additionally, the increase in travel and global trade contributes to the spread of antimicrobial resistance among countries and continents (Sridhar et al., 2021). Similar to other foodborne pathogens, a global increase in the prevalence of antibiotic resistance among *L. monocytogenes* can be seen (Ed‐Dra, 2024; Matle et al., 2019; Morvan et al., 2010). This can be due to the rapid acquisition of antibiotic‐resistant genes from other bacteria in the food processing environment (Abdollahzadeh, Ojagh, Hosseini, Ghaemi, et al., 2016; Pagadala et al., 2012; Wieczorek & Osek, 2017). Antibiotic resistance patterns differ among countries, encouraging continued monitoring (Keet & Rip, 2021). Such monitoring will also serve to prevent future risks to the human population (Basha et al., 2019).

Multidrug‐resistant (MDR) *L. monocytogenes* is concerning for public health as it could potentially complicate the treatment of listeriosis. MDR is defined as an isolate showing resistance to antibiotics in three or more different classes (Neuert et al., 2018; Schwan et al., 2021). MDR *L. monocytogenes* from fish, raw seafood and RTE seafood products have been reported (Jamali et al., 2015; Pagadala et al., 2012; Wieczorek & Osek, 2017). Jamali et al. (2015) isolated *L. monocytogenes* and *Listeria* spp. from fish markets and found that 6.8% were MDR. The *L. monocytogenes* isolates showed resistance to the antimicrobials tetracycline, ampicillin, cephalothin, penicillin G and streptomycin (Jamali et al., 2015). All the *L. monocytogenes* isolates from this study were susceptible to cefotaxime, cefuroxime, gentamycin, kanamycin and pefloxacin. Pagadala et al. (2012) reported that most of the *L. monocytogenes* isolates from blue crab and its food processing environment in the United States of America were susceptible to gentamycin, sulfamethoxazole/trimethoprim and kanamycin. Most of the isolates were also resistant to erythromycin, ciprofloxacin and tetracycline. In Korea, *L. monocytogenes* isolates from RTE seafood and the food processing environment showed the highest antibiotic resistance to benzylpenicillin, clindamycin, oxacillin, ampicillin and tetracycline. *L. monocytogenes* isolates showed low levels of resistance to the antibiotics cefoxitin, erythromycin, gentamycin, kanamycin, rifamycin, streptomycin, sulfamethoxazole/trimethoprim and vancomycin (Lee et al., 2017).

A South African study tested the antibiotic susceptibility of *L. monocytogenes* from locally sourced seafood (i.e. raw hake) (Keet & Rip, 2021). The authors found susceptibility (*p* < 0.001) of *L. monocytogenes* to all five antibiotics tested (ampicillin, chloramphenicol, erythromycin, gentamycin, and tetracycline). Another South African study found five isolates from locally sourced seafood and its processing environment (*n* = 38) to be phenotypically resistant to antibiotics, including tetracycline (*n* = 2), sulfamethoxazole/trimethoprim (*n* = 2), erythromycin (*n* = 1), and chloramphenicol (*n* = 1). One isolate showed resistance to both tetracycline and sulfamethoxazole/trimethoprim. Most isolates were susceptible to all tested antibiotics (*p* < 0.01). WGS identified genes responsible for phenotypic resistance (Lambrechts et al., 2024).

It is common to find strains recovered from food, the environment and clinical origin that show resistance to one or more antibiotics commonly employed for listeriosis treatment (Jamali et al., 2015; Pagadala et al., 2012; Wieczorek & Osek, 2017). Hence, it is crucial to track antibiotic susceptibility patterns of *L. monocytogenes* from food and the food processing environment sources across various geographic regions (Silva et al., 2024), allowing for strategies to be put in place to prevent the spread of these resistant strains.

## 6 CONCLUSION

Outbreaks of *L. monocytogenes* have been documented in seafood, with recent incidences linked to salmon and trout products, causing several fatalities. This shows the risk that seafood poses to consumers, especially those who are immunocompromised. Several studies have been done globally characterizing *L. monocytogenes* from seafood and its processing environment. Several researchers have found that serotype 1/2a is the predominant serotype followed by serotypes 4b, 1/2b, and 1/2c. CC121 and CC321 are prevalent in seafood and the seafood processing environment. Other CCs, namely CC1, CC8, CC9, CC87, and CC155, have also been identified as common in seafood products, some of which are considered hypervirulent clones.

The food‐processing environment and post‐processing activities have been shown to contribute to the spread of *L. monocytogenes*. Understanding the mechanisms of survival and persistence of these isolates within food production settings is important for enhancing food safety measures. In seafood‐related studies, the identification of sanitizer tolerance and stress survival genes may offer insight into the persistence of *L. monocytogenes* in food production facilities. When combined with antibiotic susceptibility patterns, this knowledge is crucial for safeguarding public health and may exhibit variation across different geographic regions. Examining various domains and their interactions is crucial. A research approach that encompasses clinical, food and environmental aspects enhances our comprehension of associated risks related to *L. monocytogenes* and facilitates the development of well‐informed intervention strategies.

## AUTHOR CONTRIBUTIONS

**Karlene Lambrechts**: Writing—review and editing; writing—original draft; investigation; formal analysis. **Diane Rip**: Conceptualization; funding acquisition; writing—review and editing; supervision; project administration; validation.

## CONFLICT OF INTEREST STATEMENT

None declared.

## ETHICS STATEMENT

None required.

## Data Availability

Not applicable.
